# Deployment of Critical Incident Reporting System (CIRS) in public Styrian hospitals: a five year perspective

**DOI:** 10.1186/s12913-019-4265-0

**Published:** 2019-06-24

**Authors:** Gerald Sendlhofer, Peter Schweppe, Ursula Sprincnik, Veronika Gombotz, Karina Leitgeb, Peter Tiefenbacher, Lars-Peter Kamolz, Gernot Brunner

**Affiliations:** 1 Research Unit for Safety in Health, c/o Division of Plastic, Aesthetic and Reconstructive Surgery, Department of Surgery, Medical University of Graz, Graz, Austria; 2 Executive Department for Quality and Risk Management, University Hospital Graz, Graz, Austria; 3 Department for Law and Risk Management, Styrian Hospitals Limited Liability Company (KAGes), Graz, Austria

**Keywords:** Critical incident, Incident reporting, Patient safety, Safety

## Abstract

**Background:**

To increase patient safety, so-called Critical Incident Reporting Systems (CIRS) were implemented. For Austria, no data are available on how CIRS is used within a healthcare facility. Therefore, the aim of this study was to present the development of CIRS within one of the biggest hospital providers in Austria.

**Methods:**

In the province of Styria, CIRS was introduced in 2012 within KAGes (holder of public hospitals) in 22 regional hospitals and one tertiary university hospital. CIRS is available in all of these hospitals using the same software solution. For reporting a CIRS case an overall guideline exists.

**Results:**

As of 2013, 2.504 CIRS cases were reported. Predominantly, CIRS-cases derived from surgical and associated disciplines (ranging from 35 to 45%). According to the list of hazards (also called “risk atlas”), errors in patient identification (ranging from 7 to 12%), errors in management of medicinal products (ranging from < 5 to 9%), errors in management of medical devices (ranging from < 5 to 10%) and errors in communication (ranging from < 5 to 6%) occurred most frequently. Most often, a CIRS case was reported due to individual error-related reasons (48%), followed by errors caused by organization, team factors, communication or documentation failures (34%).

**Conclusions:**

In summary, CIRS has been used for 5 years and 2.504 CIRS-cases were reported. There is a steady increase of reported CIRS cases per year. It became also obvious that disregarding guidelines or standards are a very common reason for reporting a CIRS case. CIRS can be regarded as a helpful supportive tool in clinical risk management and supports organizational learning and thereby collective knowledge management.

## Background

The Austrian healthcare care system is characterized by a high density of accessible healthcare facilities which amounts in more than 270 public and private-owned hospitals with approximately employed 23.000 physicians and more than 85.000 other healthcare professionals [1]. The Austrian social insurance system is based on compulsory insurance, solidarity and self-governance [1]. According to the “Nationwide Patient Safety Strategy for Austria” patient safety has been established in all structures and processes of healthcare systems and based on defined intervention fields the introduction and extension of a system of reporting (error reporting and learning systems) without penalties was foreseen [2].

Healthcare systems should provide the best possible diagnosis and treatment. However, where people work, there is the possibility that critical incidents or even worse, unintended errors such as medication errors occur which have the potential to result in patient harm [3, 4]. In order to increase patient safety, a so-called Critical Incident Reporting System (CIRS) was introduced in healthcare systems several years ago aiming to support the identification of potential hazards [5]. Since then, CIR systems have become one of the most widespread safety tools within and across healthcare organizations [6, 7]. CIRS opens the opportunity to report critical incidents and aims to improve patient safety in terms of making internal processes more secure [8]. So-called “near misses” i.e. critical incidents according to the definition provided by Medical Center for Quality in Medicine that affect patient safety should be reported into CIRS [9]. Furthermore, the core idea of CIRS is the reporting of own observed safety-related events, so that they can be systematically analyzed and serves the reporting person and others in the healthcare system in order to learn from them [10].

Across the world, differences emerged of how CIR-systems are used [9]. Some countries legally require the use of CIRS, while others define the use of CIRS voluntarily [10]. Therefore, apparent differences emerge as to what is being reported, which includes for example critical incidents, harm or near misses concerning employee safety [8]. Also, it came apparent that reports into CIRS include for example patient falls, needle stick injuries, technical problems or critical incidents involving healthcare professionals [8]. Thereby, it is obvious that different healthcare organizations defined their own requirements of what should be reported into their CIRS [11].

Taking the aforementioned into consideration, it is obvious that CIR systems are drastically underutilized and conversely, way over utilized [12]. These two extremes lead to further problems. Either an organization has to deal with excessive use, which makes it nearly impossible to react in a timely manner, or important information gets lost due to underreporting [11, 12]. Five key challenges of using CIRS were reported, 1) poor processing of incident reports, 2) poor engagement of consultants, 3) missing subsequent visible action, 4) inadequate funding and 5) less institutional support [13].

Apart from these challenges, there are a few more. To make CIRS an effective and supportive instrument for increasing patient safety, leadership and patient safety culture are needed [14–16]. It was shown that transformational leadership in hospitals was a significant predictor of the reporting frequency of critical incidents [1, 17, 18]. Barriers to report into CIRS may be the fear of social pressure or reprisal in cases where CIRS reports may have the potential to identify the reporting employee [7, 18, 19].

In Austria, CIRS was introduced as an non-mandatory tool only recently into the public healthcare system [9]. According to a survey performed by the Austrian Society for Quality and Safety in Healthcare, only 64.1% of all surveyed respondents used CIRS comprehensively in Austria [9]. As the aforementioned study was limited in terms of evaluating differences between Austrian provinces, authors took up the opportunity to evaluate the use of CIRS in one of the biggest hospital providers in Austria.

In the aforementioned survey it became also evident that CIR-systems are used heterogeneously in terms of what is being reported [9]. Since there are no data available concerning CIRS-deployment in terms of how often CIRS cases are reported in any Austrian hospital, it was the aim of this study to present the deployment of CIRS within one of the biggest hospital providers in Austria. Therefore, it was the aim to identify frequent types of CIRS cases as well as contributing factors to determine areas for improvements. As part of this evaluation, we also wanted to explore the hypothesis if CIRS cases increased over time and thereby gain insight if CIRS is accepted by healthcare professionals as a valid risk management tool.

## Methods

The study was approved by the Ethics Committee of Medical University of Graz (vote#: 30–028 ex 17/18). Thereafter, the system administrator for CIRS extracted data after additional permission (consent) by KAGes-Department for Law and Risk Management. Pseudonymised data were forwarded to the Executive Department for Quality and Risk Management for data analysis.

### CIRS regulation

**Fig. 1:**
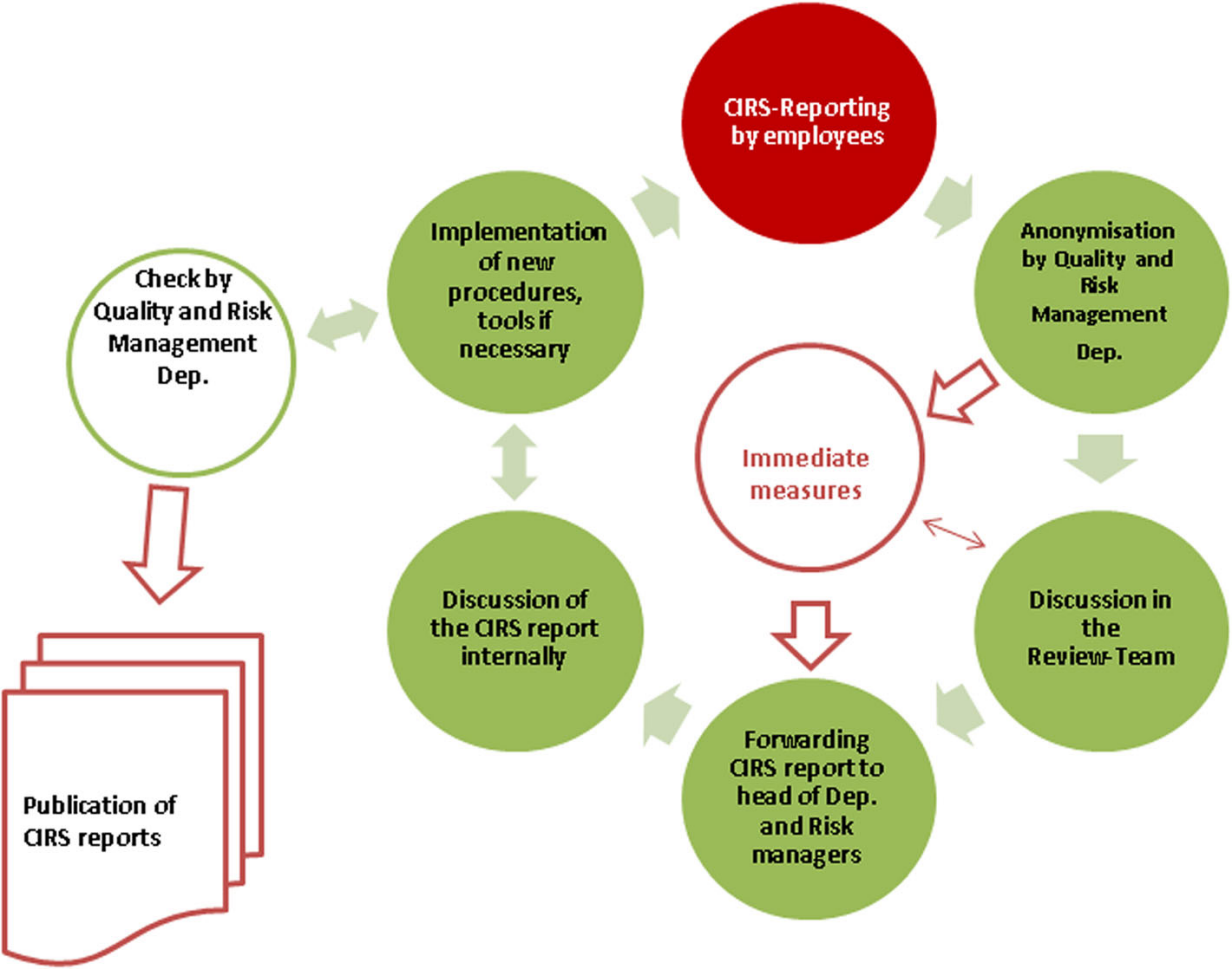
Process and decision making criteria of CIRS

In Austria, there are nine public hospital providers, one for each province, whereas KAGes is the third biggest. Within KAGes, CIRS was gradually introduced in all 23 hospitals between 2012 and 2013. CIRS is available within all these hospitals via a button on the main intranet starting homepage. Each hospital created its own CIRS button as a logo by using the same CIRS software solution (R2C by Schleupen AG©). Each hospital also prepared its own CIRS-handbook however, including the overall guideline of KAGes, which aims to advise healthcare workers on the correct use of CIRS as well as to inform them about how CIRS-cases are, processed (Fig. 1).

Within KAGes, 17.894 healthcare professionals were employed in 2017 and cared for more than 250.000 inpatients and 970.000 outpatients. Since 2013, all healthcare professionals have via the intranet-portal direct access to CIRS. Before introduction of CIRS, no baseline data were available, as healthcare professionals were not obliged to report own observed safety-related events. Only cases that caused harm to a patient were mandatory for reporting before the introduction of CIRS. However, they were not relevant for CIRS reporting according to the definition of a CIRS case.

In general, according to the CIRS-handbook harm of patients, occupational injuries, patient falls, needle stick injuries, technical reasons must not be reported into CIRS. To identify the root cause, the reporting employee has to choose from a pre-defined list the reason why a CIRS case occurred. Though technical reasons such as a breakdown of a medical device should not be reported as a CIRS case in our hospitals, contributing factors such as incorrect handling of a medical device or lack of training for a medical device should be reported. As in 2016 KAGes also implemented the reason of “physical, verbal or aggressiveness and attacks of patients”, the original CIRS tool which focused on patient safety was extended to also support employee safety. Furthermore, the reporting healthcare professionals has also the opportunity to choose from a dropdown menu where the CIRS case occurred (e.g. ward, endoscopy, outpatient ward, operating theatre, intensive care unit, laboratory, etc.). If one of the aforementioned CIRS case was reported into CIRS, the anonymizer will not approve the CIRS case of being electronically forwarded to the review team. An independent person first checks incoming CIRS-cases in each hospital if the reported case is anonymous and if not, the CIRS-case is anonymized. Then the CIRS-case is forwarded electronically to a so-called review team, which consists of experts such as physicians, nurses, quality and risk managers or jurists. Jurists are part of the review team in the university hospital as they screen any CIRS case in terms of a potential harm. If so, the CIRS case will not be discussed in the review team. They discuss each case in a timely manner in each hospital and try to deduce solutions for the reported case. The problem-solving approach together with the reported CIRS case is then forwarded to the reporting department, which is the only known identifier of a reported CIRS-case. The respective department is then obliged to discuss the CIRS case with in-house clinicians with and without managerial functions (interdisciplinary management, quality and risk managers of the department). They clarify if the suggested problem-solving approach from the review team can be transformed into daily routine in order to help to minimize the potential risk of the reported critical incident. Each CIRS case with the problem-solving approach is then published in the Intranet of the respective hospital, so that other healthcare professionals can learn from already reported CIRS cases. Within our company, only critical incidents should be reported into CIRS, meaning those cases, which did not actually cause any damage, but have the potential to cause damage if no intervention occurs.

### CIRS intranet tool

The following data is required of the reporting healthcare worker: date of CIRS reporting, department, what has happened, reporting healthcare profession, how did the critical incident emerge, cause with predefined reasons (individual-related reasons, patient-related reasons, organization/team factor/communication/documentation and medical device associated reasons).

For the evaluation of the deployment of the non-mandatory CIRS all available date from the CIRS were analyzed. Each CIRS case was categorized according to a list of hazard, which allowed clustering of CIRS-cases into several risk categories including patient rights, employee tasks, organization of patient care, patient care as such, technical issues or facility management). Voluntarily items with pre-defined drop-down menus were: where, whereby and when did it happen, on which day did it happen and how fast did the critical incident emerge.

### Statistics

There was one source of data for this study. All data were extracted by the system administrator for CIRS from the database (R2C by Schleupen AG©) for all participating hospitals within KAGes after receipt of the positive ethical vote. Data were pseudonymised, so that no conclusions were possible for individual participating hospitals or healthcare professionals within KAGes. According to pre-defined data fields, we identified the professional disciplines, number of reported CIRS cases, categories as well as reasons for CIRS cases for the years 2013 to 2018. All data were analyzed with respect to frequency distribution and proportion (%). All analyses were conducted using R version 3.4.2.

## Results

Since the introduction of the non-mandatory CIRS, in total, 2.504 CIRS cases were reported during the observational period between 2013 and 2017 (2013: *n* = 295; 2014: *n* = 471; 2015: *n* = 435; 2016: *n* = 585; 2017: *n* = 718). Thereof, CIRS-cases were derived predominately from surgical departments, anesthesiology and intensive care units (2013: *n* = 122 (42%); 2014: *n* = 164 (35%); 2015: *n* = 165 (38%); 2016: *n* = 207 (35%); 2017: *n* = 322 (45%)) (see also Table 1).

**Table 1:** CIR-cases categorized according to professional disciplines (n/%)

Year	N reports	Anesthesiology	Surgical disciplines	Non-surgical disciplines	Inter-disciplinary^a^	Administration	Others
2017	718 (29%)	103 (14%)	219 (31%)	121 (17%)	25 (3%)	25 (3%)	225 (32%)
2016	585 (23%)	52 (9%)	155 (26%)	115 (20%)	28 (5%)	12 (2%)	223 (38%)
2015	435 (17%)	48 (11%)	117 (27%)	90 (21%)	21 (5%)	7 (2%)	152 (34%)
2014	471 (19%)	55 (12%)	109 (23%)	113 (24%)	17 (4%)	4 (1%)	173 (36%)
2013	295 (12%)	37 (13%)	85 (29%)	60 (20%)	12 (4%)	6 (2%)	95 (32%)
Total	2.504 (100%)	295 (12%)	685 (26%)	499 (21%)	103 (4%)	54 (2%)	868 (35%)

^a^Interdisciplinary = i.e. involvement of radiology in a critical incident

According to Table 2, nursing staff, followed by physicians and medical-technical services (MTS), reported the majority of CIRS-cases. Most commonly, the critical incident was detected because of personal attention (41%), followed by routine checks (13%) and at random (11%) (see also Table 2). By classification of CIRS cases according to the KAGes list of hazards, the following CIRS cases where reported most often between 2013 and 2017 (including > 5% of reported CIRS cases per year): errors in patient identification (2013: 12%; 2014: 11%; 2015: 10%; 2016: 11%; 2017: 7%), errors in management of medicinal products (2013: 9%; 2014: 6%; 2015: 5%; 2016: 5%; 2017: < 5%), errors in management of medical devices (2013: 10%; 2014: 6%; 2015: 5%; 2016: < 5%; 2017: < 5%) and errors in communication (2013: < 5%; 2014: 6%; 2015: 6%; 2016: 5%; 2017: 5%). Furthermore, below the level of 5%: errors in clinical documentation, errors in diagnostics, and errors in surgical procedures were mentioned as reasons for reporting a CIRS case.

**Table 2:** 2.504 CIR-cases categorized according to reporting professional disciplines and how a CIRS emerged (n/%)

Year	Physician	Nursing	MTS^a^	Others	Routine check	Personal attention	At random	Others
2017	149 (21%)	443 (61%)	71 (10%)	55 (8%)	97 (14%)	286 (40%)	59 (8%)	276 (38%)
2016	98 (17%)	352 (60%)	84 (14%)	51 (9%)	74 (13%)	247 (42%)	68 (12%)	196 (33%)
2015	108 (25%)	227 (52%)	64 (15%)	36 (8%)	65 (15%)	187 (43%)	56 (13%)	127 (29%)
2014	118 (25%)	278 (59%)	51 (11%)	24 (5%)	67 (14%)	182 (39%)	49 (10%)	173 (36%)
2013	96 (33%)	134 (46%)	35 (12%)	30 (9%)	32 (11%)	125 (42%)	45 (15%)	93 (31%)
Total	569 (23%)	1.434 (57%)	305 (12%)	196 (8%)	335 (13%)	1.027 (41%)	277 (11%)	865 (35%)

^a^*MTS* Medical technical assistant

Most often, a CIRS case was reported due to individual error-related reasons (48%), followed by errors caused by organization, team factors, communication or documentation failures (34%) and medical device associated reasons (10%) (see also Table 3).

**Table 3:** CIR-cases categorized according to cause with predefined reasons (n/%), for each category multiple answers were possible

Year	Individual related reason	Organization/team factor/communication/documentation	Medical device associated reasons	Patient related reason
2017	940 (48%)	627 (32%)	245 (12%)	165 (8%)
2016	722 (48%)	457 (30%)	201 (13%)	136 (9%)
2015	598 (47%)	455 (36%)	118 (9%)	102 (8%)
2014	635 (48%)	493 (37%)	103 (8%)	86 (7%)
2013	395 (50%)	287 (36%)	59 (7%)	50 (7%)
Total	3.290 (48%)	2.319 (34%)	726 (10%)	539 (8%)

According to predefined causes and reasons within the CIRS, individual-related reasons, lack of knowledge, failure in process planning, disregarding guidelines or standards and negligence were mentioned most often. CIRS cases caused by organization, team factors, and communication or documentation failures were predominantly caused by poor communication in and between occupational groups, deficiency in documentation, poor coordination and failure in process planning. Medical device associated reasons were handling of medical devices, insufficient introductory training, defective work equipment and insufficient construction measures. As to patient-related reasons very ill patients, communication problems, acute change of illness and since 2016 physical, verbal or aggressiveness and attacks of patients were reported (see Table 4).

**Table 4:** CIR-cases categorized according to cause with predefined reasons (n/%)

Year	2013	2014	2015	2016	2017
Cause due to					
Individual related reason					
- Lack of knowledge	42 (11%)	68 (11%)	58 (10%)	95 (13%)	129 (14%)
- Failure in process planning	40 (10%)	67 (11%)	49 (8%)	54 (7%)	72 (8%)
- Fisregarding guidelines or standards	67 (17%)	120 (19%)	134 (22%)	152 (21%)	157 (17%)
- Lack of attention	83 (21%)	116 (18%)	109 (18%)	144 (20%)	164 (17%)
Organization, team factor, communication, documentation reasons					
- Poor communication in one occupational group	35 (12%)	51 (10%)	44 (10%)	42 (9%)	61 (10%)
- Poor communication between occupational groups	54 (19%)	78 (16%)	79 (17%)	77 (17%)	85 (14%)
- Deficient documentation	33 (11%)	52 (11%)	32 (7%)	49 (11%)	59 (9%)
- Poor coordination	28 (10%)	43 (9%)	47 (10%)	42 (9%)	61 (10%)
Medical device associated reasons					
- Handling of medical devices	51 (86%)	86 (83%)	101 (86%)	77 (38%)	83 (34%)
- Insufficient introductory training	8 (14%)	17 (17%)	17 (14%)	13 (6%)	22 (9%)
- Defective work equipment	–	–	–	30 (15%)	45 (18%)
- Insufficient construction measures	–	–	–	48 (24%)	53 (22%)
Patient related reason					
- Very ill patients	29 (58%)	46 (53%)	51 (50%)	30 (22%)	37 (22%)
- Communication problems	10 (20%)	7 (8%)	8 (8%)	21 (15%)	15 (9%)
- Acute change of illness	8 (16%)	22 (26%)	32 (31%)	31 (23%)	43 (26%)
- Physical, verbal or aggressiveness and attacks of patients	0 (0%)	0 (0%)	0 (0%)	36 (26%)	34 (21%)

## Discussion

Most hospitals have installed systems to collect quality and safety data, within KAGes 2.504 CIRS cases were reported on a voluntarily basis by healthcare professionals in 22 regional and one tertiary university hospital over a period of 5 years. During this observational period, the number of CIRS cases increased steadily from 295 reports in 2013 to 718 reports in 2017. Although there is a continuous progress of reported CIRS cases recognizable, we assume that the amount of 2.504 CIRS cases only represent a small fraction of all available critical incidents. Is the amount of reported CIRS-cases an indicator for a good or a bad safety culture?

As to the question regarding the amount of reported CIRS-cases, a comparison of different countries with different approaches is necessary. In mature CIRS-healthcare organizations, for example NHS, more than 1 million CIRS cases are reported each year into the mandatory system [20]. However, in order to address the aforementioned question conclusively, certain different framework conditions have to be considered between a German-speaking and others. First of all, according to a survey, CIRS has not yet been extensively introduced in Austrian healthcare organizations [9]. Furthermore, the Austrian healthcare system provides many different possibilities in order to report potential improvements. For example, there is a register of fall incidents or falls are documented in a separate file in the respective hospital information system, needle stick injuries are documented by representatives of occupational medicine and technical issues are part of an immediate reporting to the technical experts [9]. Therefore, employees are particularly challenged by the number of different reporting systems to select the right instrument. It should also be considered whether different reporting systems could be standardized to a certain extent. At the very least in such a way that the reporter has only one reporting portal (CIRS, Near Miss, Idea Management, etc.) and that the underlying processing paths are regulated by a single defined workflow. In any case, the user-friendliness should be increased. In order to rate the number of KAGes CIRS-reports in the context of Austrian healthcare facilities, relevant data are missing so far. There is no comprehensive overview of other healthcare organizations in Austria, if a CIRS is implemented, how CIRS is used and if it is used, which results and conclusions can be drawn.

Comparing our results with Swiss data who also use CIRS as a non-mandatory system, the University Hospital Zurich reported 1.400 CIRS cases within 1 year [21]. In this context it has to be considered that CIRS was launched in Zurich before 2012. According to the publically available NHS CIRS-statistics, healthcare professionals reported 43.767 CIRS-cases in the second year of introduction of CIRS in England and Wales. Extrapolating the numbers of CIRS cases in our company in relation to the number of NHS hospitals, an approximately comparable amount occurred for the year 2004 within KAGes. However, these NHS CIRS-reports included fall or sentinel incidents, which in our case had been excluded of CIRS. Nevertheless, in NHS CIRS-numbers increased exponentially in subsequent years.

Within KAGes, healthcare experts were made aware of CIRS in repetitive trainings, newsletters and conferences, which probably supported the steady increase of CIRS reports per year during the observational period. When healthcare experts were interviewed in an unstructured way about their knowledge of CIRS, their most common answer was that CIRS is a well-known risk management tool. Regarding the use of CIRS, there was hardly any knowledge. They also explained why they do not use it: First, they do not think about reporting a certain case to CIRS due to the daily workload and further documentation responsibilities. Secondly, if anything nearly happened they fixed it immediately and did not think that others could probably also profit from their near miss and learn from CIRS cases. Thirdly, anonymity is still a topic. Though it is clearly expressed by the hospital provider that anonymity is guaranteed, healthcare experts are afraid of reporting a CIRS-case electronically via their personal computer account. Finally, many healthcare experts still have the fear that a case can be de-anonymized by their colleagues during the process of problem solving within a department, which could also be interpreted as a low patient safety culture*.*

Within KAGes, nurses most commonly reported CIRS-cases; however, the number of reporting physicians is steadily increasing. We assume that continuous training and information on using CIRS needs a certain period to attract all healthcare professionals.

According to Welters et al. major categories for critical incidents were 1) equipment, 2) clinical practice, 3) pharmaceuticals, 4) administration and 5) health & safety hazards [22]. Within our hospitals, two major predefined categories were mentioned, mainly individual-related reasons and organization/team factors, communication and documentation. Communication deficits in terms of verbal, written or other kind of communication are among the most important causes for critical incidents [6, 23]. Within individual reasons, lack of attention and non-adherence to guidelines (negligence) were expressed most commonly. Between 17 to 22% neglected existing guidelines or standards. Examples for negligence where missing or wrong usage of the surgical safety checklist, illegibility of prescriptions or missing hand hygiene for which internal and international guidelines do exist [24–26]. Failure to comply with guidelines may be because there are too many of them or that these guidelines are not trained adequately. Despite others forced to implement guidelines in order to overcome patient safety risks [22], our data demonstrated that disregarding guidelines or standards are a very common cause for a critical incident. This is a particular problem, especially in a university hospital, where the proportion of trainees is particularly high and in parallel, there are a high numbers of trainee turnovers every year. The topic of lack of attention is a very important factor and it is a sign of stress, rush, routine procedures or ignorance. In this sense, one of the main tasks in the near future is to sharpen the awareness and to draw particular attention to these hazards (keyword “human factor”). It was also obvious that poor communication between healthcare professional groups played a major role concerning the occurrence of critical incidents [23].

The categorization of CIRS cases according to the KAGes list of hazards and predefined reasons already helped a lot to identify areas of action within the entire company. Therefore, CIRS can be regarded as a helpful and supportive tool in clinical risk management to get aware of certain risks that may lead to improvements within the reporting organization. Organizational learning by using CIRS and thereby collective knowledge are prerequisites for succeeding in patient safety. Therefore CIRS should be promoted in every healthcare organization [27].

This is the first report of one of the biggest hospital providers in Austria aiming to gain insight into their use of CIRS. This study has several strengths and limitations. First, it can be drawn from the presented data that its healthcare professionals increasingly use CIRS. Secondly, data already shows areas for improvement; therefore, CIRS can be regarded as a helpful tool for collective knowledge management. A major limitation is the lack of comparable data within the Austrian healthcare system and between other hospital providers. Nevertheless, self-reporting systems such as CIRS carries the risk of underreporting. Lastly, within this study it was not investigated whether the reporting led to some meaningful changes in practices so far.

## Conclusions

To sum up, it can be stated that CIRS has been used within KAGes for 5 years and 2.504 CIRS cases were reported on a voluntarily basis by healthcare professionals. It was recognizable that there was a steady increase of reported CIRS cases per year. In Austria, reporting of CIRS cases is not comparable to healthcare organizations such as the NHS. Nevertheless, CIRS can be regarded as a helpful supportive tool in clinical risk management and may support organizational learning and thereby collective knowledge management using it as a non-mandatory system. Knowledge of CIRS types in terms of categories and reasons can facilitate organizational improvement. Our results suggest that analysis of CIRS types and frequency is crucial to raise awareness. It became also obvious that disregarding guidelines or standards are a very common reason for reporting a CIRS case. Therefore, much emphasis was put in place to improve compliance for topics such as hand hygiene, legibility and completeness of handwritten prescriptions or using the surgical safety checklist [24–26]. Further research is necessary to understand the major drivers and the impact of CIRS on patient safety.

## Data Availability

The authors confirm that all data underlying the findings are fully available without restriction. The datasets used and/or analysed during the current study are available from the corresponding author on reasonable request.

## Abbreviations

CIRS: Critical incident reporting systems
CIR-system: Critical incident reporting system
